# Supplementary material for: Floquet-tailored Rydberg interactions
Source: Nat Commun. 2023 Nov 6;14:7128. doi: 10.1038/s41467-023-42899-8 (PMC10628180; doi:10.1038/s41467-023-42899-8)
Supplement: Supplementary file 1 — Supplementary Information [file 41467_2023_42899_MOESM1_ESM.pdf]

Supplementary Information: Floquet-Tailored Rydberg Interactions

Luheng Zhao,[1] Michael Dao Kang Lee,[1] Mohammad Mujahid Aliyu,[1] and Huanqian Loh[1,2,*]

[1]*Centre for Quantum Technologies, National University of Singapore, 117543 Singapore, Singapore*
[2]*Department of Physics, National University of Singapore, 117542 Singapore, Singapore*

SUPPLEMENTARY NOTE 1: STATE PREPARATION AND MEASUREMENT ERRORS

The ground and Rydberg states are distinguished by the atom survival and loss after the second imaging pulse is applied during the experiment sequence. However, the probabilities of detecting the ground and Rydberg states (P_g, P_e) are susceptible to false positive errors ϵ and false negative errors ϵ', which can obscure the true probabilities $(\tilde{P}_g, \tilde{P}_e)$.

False positive errors $\epsilon = P(e|\tilde{g})$ refer to the case where lost ground state atoms are misinterpreted as Rydberg atoms. The loss of ground state atoms can occur due to background collisions, the atom-recapture inefficiency, and the heating of ground state atoms during imaging. In our experiment, the last mechanism dominates the false positive error, which is measured to be 0.03. We note in passing that while the D_1 imaging survival probability in this experiment is 0.97, it can generally be higher (0.99) if the imaging configuration previously reported in[1] is used.

On the other hand, false negative errors $\epsilon' = P(g|\tilde{e})$ arise from Rydberg atoms that have decayed to the ground state and are recaptured before exiting the tweezer capture range. These atoms are consequently miscounted as ground state atoms. In contrast, atoms that remain in the Rydberg state would be repelled by the anti-trapping potential of the tweezer. For a 1.3 mK recapture trap depth, the estimated time required for Rydberg atoms to leave the trapping region is 4.5 μs. Given the natural lifetime of 260 μs for the $59S_{1/2}$ Rydberg state[2], we expect a false negative error of 0.02. In addition, before the tweezer light is turned back on, there is a delay of 0.5 μs during which only the 409 nm Rydberg laser is left on. Combined with the off-resonant scattering rate of the 409 nm laser, this delay adds 0.01 to the false negative error. Therefore the total false negative error is 0.03.

The detected and true populations are then related to each other by the following expressions[3]:

$$P_g = \eta(1-\epsilon) + (1-\eta)(1-\epsilon)(\tilde{P}_g + \epsilon'\tilde{P}_r), \quad (1)$$

$$P_r = \eta\epsilon + (1-\eta)(\epsilon\tilde{P}_g + (1-\epsilon'+\epsilon\epsilon')\tilde{P}_r)\,, \quad (2)$$

where η is the error associated with imperfect optical pumping and is estimated to be 0.009 in our experiment. For the two-atom populations, the above probabilities are multiplied accordingly.

* phylohh@nus.edu.sg

SUPPLEMENTARY NOTE 2: CALIBRATION OF ATOM SEPARATION

Precise control of the separation distance between atoms is important as it determines the strength of interaction between the atoms when excited to Rydberg states. In the FFM studies, the nominal atom spacing ranges from 5.6 μm to 10.3 μm. The spacing is controlled by the frequencies sent to the acousto-optic deflector (AOD). Calibration of the AOD frequency spacing is performed by directly measuring the interaction-induced energy shift experienced by two atoms in the $|ee\rangle$ state with the following sequence: first, the two atoms are excited from $|gg\rangle$ to $|W\rangle$ with a resonant monochromatic π-pulse ($\delta = 0$); subsequently, $|W\rangle$ is excited to $|ee\rangle$ with a second monochromatic pulse of variable detuning δ'. The $|ee\rangle$ population is maximized when $\delta' = V$. The above sequence is repeated at different AOD frequency spacings. The measurements are fit with the calculated value[4] of C_6 for the $59S_{1/2}$ Rydberg state of ^{23}Na, $C_6 = 2\pi \times 251.288$ GHz μm^6, and an overall distance-independent frequency offset δ_u arising from an imperfection in determining the unshifted resonance (Supplementary Fig. 1). The fit distance calibration of 0.780(2) μm/MHz is consistent with the expected value (0.783 μm/MHz) calculated from the acousto-optic deflector specifications, objective focal length, and tweezer telescope magnification.

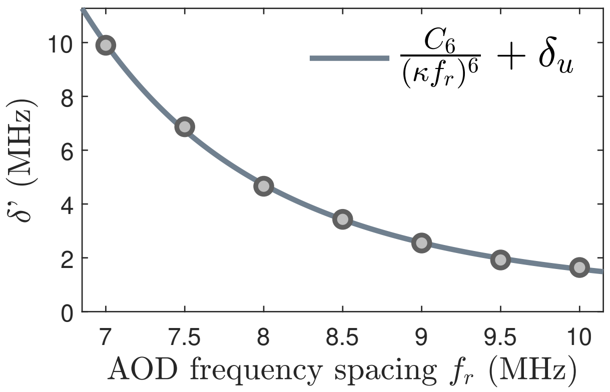

Supplementary Figure 1. **Calibration of the interatomic distance r with respect to the AOD frequency spacing f_r.** Error bars are smaller than the displayed marker sizes. The calibration factor κ is determined to be 0.780(2) μm/MHz.

SUPPLEMENTARY NOTE 3: FFM DETAILS

The FFM is implemented by sending the 589 nm Rydberg laser through an acousto-optic modulator (AOM), which is driven by an arbitrary waveform generator (AWG) to yield a time-varying frequency ($-\delta \sin \omega_0 t$). We ensure that the frequency-modulated RF signal has a well-defined phase by triggering the AWG. The AOM is used in a double-pass configuration so that the output beam angle sent to the neutral atom array remains unchanged despite the time-varying single-pass diffraction angle. To enable a fast on-off response of the AOM, we focus the 589 nm Rydberg excitation laser to a beam waist of 35 μm in the AOM.

Calibration of modulation index

Since the dynamics of the two-atom system depends on the modulation index α, it is important to calibrate the modulation amplitude δ, which can differ from the AWG-specified amplitude due to the finite AOM bandwidth. This is performed by monitoring an optical beat note between the first-order deflected beam with a reference beam. For a given applied modulation frequency ω_0, we measure the power of the beat note carrier while changing the modulation amplitude δ. The normalized carrier power measured with respect to the modulation index α is fitted to the modulus of the zeroth order Bessel function with a free linear scaling factor χ (Supplementary Fig. 2a, b). We find that the AWG-specified modulation index is slightly larger than the theoretical value by $\chi = 1.009(2)$ and 1.0450(9) for ω_0 = 3 MHz and 6 MHz, respectively. This means that the actual modulation index effected by the AOM is correspondingly smaller than the AWG-specified value. We note that χ is sensitive to the laser beam alignment through the AOM.

To further investigate the effect of the modulation frequency on χ, we measure the first Bessel function zero α_0 (i.e. where $J_0(\alpha_0) = 0$) for different modulation frequencies by tracking the modulation amplitude at which the carrier power is minimized. Similarly, we observe the measured modulation index to be higher than the theoretical Bessel zero of 2.40. The finite AOM bandwidth yields a larger deviation for higher modulation frequencies, as shown in Supplementary Fig. 2c.

Minimization of residual amplitude modulation

Having a residual amplitude modulation (RAM) is undesirable as it would change the dynamics from that predicted by the Hamiltonian given in:

$$\frac{H}{\hbar} = -\Delta(t) \sum_{i=1}^{N} \sigma_{ee}^{i} + \frac{\Omega}{2} \sum_{i=1}^{N} \sigma_{x}^{i} + \sum_{i<j} V_{ij} \sigma_{ee}^{i} \sigma_{ee}^{j} \,, \tag{3}$$

In particular, the entanglement coherence will be impacted most significantly as the dynamical freezing of $|W\rangle$ is sensitive to the ability to precisely zero the two-atom Rabi frequency Ω_a. We model the RAM as a modification of the Rabi frequency between the $|gg\rangle$ and $|W\rangle$ states from

$$\Omega_a(t) \propto \Omega \sum_{m=-\infty}^{\infty} J_m(\alpha) e^{im\omega_0 t + im\frac{\pi}{2}} \tag{4}$$

to

$$\begin{aligned} \Omega_a \;&\rightarrow\; \Omega \left[1 + \sum_{n=1}^{\infty} (A_n \sin n\omega_0 t + B_n \cos n\omega_0 t)\right] \sum_{m=-\infty}^{\infty} J_m(\alpha) e^{im\omega_0 t + im\frac{\pi}{2}} \\ &=\; \Omega \left[1 + \sum_{n=1}^{\infty} (A_n \sin n\omega_0 t + B_n \cos n\omega_0 t)\right] \left[J_0(\alpha) + \sum_{m=1}^{\infty} J_m(\alpha) e^{im\frac{\pi}{2}} \cos m\omega_0 t\right] \end{aligned} \tag{5}$$

where A_n and B_n are assumed to be small. Supplementary Eq. (5) shows that to lowest order, only the cosine components of the RAM would perturb $J_0(\alpha)\Omega$, whereas the sine components of the RAM (i.e. components in phase with FFM) exert minimal effect on Ω_a.

The RAM can arise either from our modulation of the single-photon detuning Δ', which leads to an effective modulation of the two-photon Rabi frequency Ω, or from the frequency-dependent diffraction efficiency of the AOM. For the former, we estimate an effective modulation of $\delta/\Delta' =$ 4% on Ω for the experiments involving the largest modulation indices. However, since the modulation on Ω is in phase with the FFM, we did not attempt to compensate for this factor. On the other hand, the RAM arising from the AOM imperfection is minimized in our experiment by either calibrating the frequency-dependent diffraction efficiency or combining the calibration with negative feedback based on gradient descent.

For the calibration, a quadratic correction factor was initially applied to the AOM:

$$V_{\text{AOM}} \rightarrow \frac{V_{\text{AOM}}}{a(f - f_c)^2 + c} \,, \tag{6}$$

where the coefficients a, f_c, and c were obtained by minimizing the power variation as the frequency of the AOM was linearly ramped. We then conducted a more detailed calibration of the AOM diffraction efficiency by fitting the output power

to the following function:

$$P(f, V_{\rm AOM}) = A(f) \tanh\left(\frac{V_{\rm AOM} - V_0(f)}{\sigma(f)}\right) + C(f)\,, \quad (7)$$

where the frequency-dependent fitting parameters $A(f)$, $V_0(f)$, $\sigma(f)$, and $C(f)$ were in turn determined by a polynomial fit to the frequency. To obtain a constant power output P independent of f, we invert the expression to get the AOM amplitude required:

$$V_{\rm AOM}(P, f) = V_0(f) + \sigma \tanh^{-1}\left(\frac{P - C(f)}{A(f)}\right)\,. \quad (8)$$

Through this detailed calibration, we can reduce the power output error from ≈30% peak-to-peak variation to <10% peak-to-peak variation when we manually change the frequency tones over a 50 MHz bandwidth. However, when we perform FFM, we observe that the calibration only reduces the DC offset error (Supplementary Fig. 2e), while there is still an error that is synchronous with the frequency modulation.

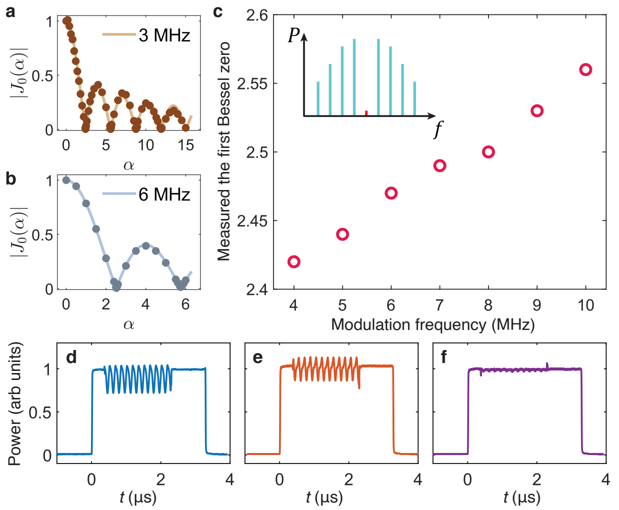

Supplementary Figure 2. **Mitigation of FFM imperfections. a, b** Calibration of modulation index for **a** $\omega_0 = 3$ MHz and **b** $\omega_0 = 6$ MHz, by measuring the normalized carrier power versus AWG-specified modulation index α. The measured data is fit to $|J_0(\alpha)|$. **c** Calibration of modulation index for $\omega_0 = 4 - 10$ MHz, performed by measuring the positions of the Bessel function zeros α_0, where $J_0(\alpha_0) = 0$. (Inset) Schematic of the optical beat note power spectrum, where the (red) carrier is minimized at α_0. **d** Measured Rydberg laser excitation pulse with FFM ($\omega_0 = 6$ MHz, $\alpha = 5.5$) and no amplitude compensation. **e** Measured Rydberg laser excitation pulse with the same FFM parameters, incorporating a calibration of the AOM frequency-dependent diffraction efficiency. **f** Measured Rydberg laser excitation pulse with same FFM parameters and both calibration and gradient descent compensation.

To further mitigate the RAM, we perform an additional feedback process. We iteratively add a set of n harmonics of the modulation frequency, where $n = 1$ – 4 with both quadratures (sine and cosine) applied. The amplitudes of these harmonics are varied using gradient descent. Supplementary Fig. 2f shows a minimized RAM of 2.2% when both calibration and gradient descent optimization methods are employed.

SUPPLEMENTARY NOTE 4: NUMERICAL SIMULATION OF DYNAMICS

For our numerical modeling, we use the Python package QuTiP[5] to simulate the dynamics as governed by the Lindblad master equation[3,6,7]:

$$\dot{\rho} = -\frac{i}{\hbar}[H, \rho] + \mathcal{L}[\rho]\,. \quad (9)$$

$\mathcal{L}[\rho]$ is a sum of the following three terms corresponding to off-resonant scattering from the intermediate state $\mathcal{L}_m$, spontaneous decay from the Rydberg state $\mathcal{L}_r$, and laser phase noise $\mathcal{L}_l$, respectively:

$$\mathcal{L}_m[\rho] = \sum_i (L_m^i \rho (L_m^i)^\dagger - \frac{1}{2}\{(L_m^i)^\dagger L_m^i, \rho\})\,, \quad (10)$$

$$\mathcal{L}_r[\rho] = \sum_i (L_r^i \rho (L_r^i)^\dagger - \frac{1}{2}\{(L_r^i)^\dagger L_r^i, \rho\})\,, \quad (11)$$

$$\mathcal{L}_l[\rho] = L_l \rho L_l^\dagger - \frac{1}{2}\{L_l^\dagger L_l, \rho\}\,, \quad (12)$$

where i indexes the atom.

The off-resonant scattering can arise from both the 589 nm Rydberg laser γ_1 and the 409 nm Rydberg laser γ_2, which are described by their corresponding Lindblad operators $L_m^{(i)} = \sqrt{\gamma_1}|g_i\rangle\langle g_i| + \sqrt{\gamma_2}|g_i\rangle\langle e_i|$. In the experiment, $\gamma_1 \approx 2\pi \times 17$ kHz and $\gamma_2 \approx 2\pi \times 2.4$ kHz. The blackbody-radiation-limited lifetime[2] of the $59S_{1/2}$ Rydberg state is $1/\Gamma_r = 106.5$ μs and is modeled with the simplified Lindblad operator $L_r^{(i)} = \sqrt{\Gamma_r}|g_i\rangle\langle e_i|$. Finally, the laser phase noise γ_l is modeled as a global dephasing term: $L_l = \sqrt{\gamma_l/2}\,\sigma_z^{(1)} \otimes \sigma_z^{(2)}$. γ_l is inferred from the coherence time of the single-atom Rabi oscillation between $|g\rangle$ and $|e\rangle$ to be $2\pi \times 50$ kHz.

The calculated populations are then scaled to include the state preparation and measurement errors described in Section .

At a temperature of 1.2 μK, the atoms have a finite radial and axial position spread of $\{\sigma_{x,y}, \sigma_z\} \approx \{0.17$ μm, 0.92 μm$\}$ in the tweezers, which increase slightly when the tweezers are turned off during the Rydberg excitation. We model this effect by using Monte Carlo methods to randomly sample the atom position from a normal distribution determined by its initial position uncertainty. We then take into account the effect of the finite velocity, which changes the atom spacing over time. The Doppler shifts for each atoms are also randomly sampled from normal distributions. The simulated two-atom populations are sorted into histograms, of which the standard deviations are depicted as shaded curves in Figs. 2c inset, 2d, 2e, 3b, 3c, and 4c of the main text. In Fig. 2c (main plot) of the main text, the Monte Carlo sampling is not depicted for clarity. As a sanity check, Supplementary Fig. 3 shows the observed two-atom dynamics driven by a static Rydberg excitation scheme, where $V = 6\ \Omega$ and where the measured populations are in good agreement with those from the Monte Carlo simulations with the above Lindblad terms.

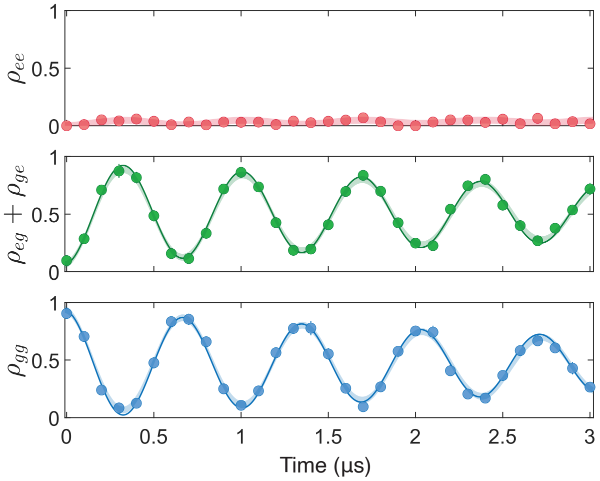

Supplementary Figure 3. **Two-atom dynamics under static Rydberg excitation with $V = 6\ \Omega$.** Dynamics of (top) ρ_{ee}, (middle) $\rho_{eg} + \rho_{ge}$, and (bottom) ρ_{gg}. The markers indicate the experimental measurements, which are fit (solid line) to yield a collective Rabi frequency of $2\pi \times 1.466(3)$ MHz and a 5.0(6) μs decay time constant. The shading reflects the Monte Carlo simulation results.

In Fig. 4 of the main text, we note that the $|ee\rangle$ population at short times (Fig. 4c) can be boosted by using a less aggressive ramp of the trap depth, since the interaction disorder there mainly comes from the initial position spread. Where we consider the anti-blockade dynamics under ground-state cooling, we assume a final motional state occupation of $\{\bar{n}_{x,y}, \bar{n}_z\} = \{0.05, 0.20\}$. We further assume that the atoms can remain trapped during the Rydberg excitation, such that there is no further increase in the position uncertainty due to time-of-flight dynamics. For the idealized scenario combining ground-state cooling with enhanced coherence (Figs. 4c, d of the main text), we assume suppressed off-resonant scattering rates and a phase noise of $2\pi \times 1$ kHz for each laser. In addition, we assume working in the $n = 80$ Rydberg state of ^{23}Na, which offers a longer lifetime[2]. The combined suppression of decoherence effects would increase the effective two-atom coherence time to 74 μs. For these idealized cases, the corresponding plots in Figs. 4c and 4d of the main text do not include state preparation and measurement (SPAM) errors.

SUPPLEMENTARY NOTE 5: FIDELITY ESTIMATION

To estimate the fidelity of the $|W\rangle$ state generated in the enhanced blockade regime, we first solve the Lindblad master equation over the excitation time t to yield the density matrix $\rho^{(j)}(t)$ for a given set of random initial atom positions and velocities. We use the same initial positions $\{x_1^{(j)}, x_2^{(j)}\}$ to define the symmetric state[6]:

$$|W^{(j)}\rangle = e^{ikx_1^{(j)}} \left(|ge\rangle + e^{ik(x_2^{(j)} - x_1^{(j)})} |eg\rangle \right), \tag{13}$$

where j indexes the set of initial conditions and k is the effective wavevector of the two counterpropagating Rydberg lasers. We note that the relative phase $k(x_2^{(j)} - x_1^{(j)})$ is nominally fixed for each experiment trial. We calculate the fidelity $\mathcal{F}^{(j)}(t) = \langle W^{(j)} | \rho^{(j)}(t) | W^{(j)} \rangle$ as a function of time, while taking note of the maximum fidelity $\mathcal{F}^{(j)}_{\max}$ achieved during the excitation period. The above procedure is repeated for different sets of initial conditions randomly sampled from their respective normal distributions as described in Section . Supplementary Fig. 4 shows the calculated fidelities $\mathcal{F}^{(j)}(t)$ for different initial conditions corresponding to the data shown in Fig. 2e of the main text. The reported fidelity of 0.77(5) is the mean and standard deviation of $\mathcal{F}_{\max}$.

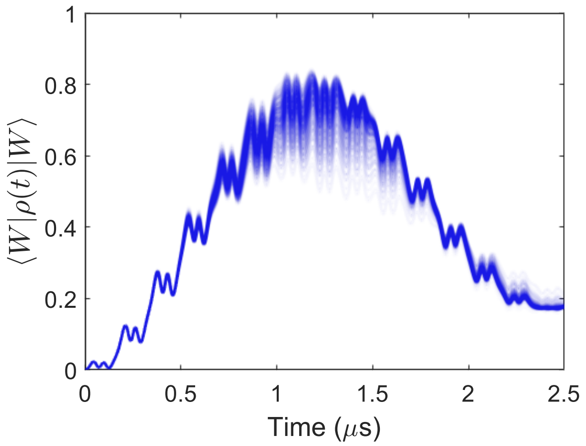

Supplementary Figure 4. **Calculated fidelities $\langle W|\rho(t)|W\rangle$.** The time evolution of the density matrix $\rho(t)$ is obtained from numerically modeling the data shown in Fig. 2e of the main text, where $V = 0.8\ \Omega, \omega_0 = 3\ \Omega$, and $\alpha = 6.9$. The different curves indicate various initial atom positions and velocities randomly sampled from normal distributions, whose standard deviations correspond to an atom temperature of 1.2 μK.

SUPPLEMENTARY NOTE 6: ENHANCED QUBIT CONNECTIVITY

The $|W\rangle$ fidelity of 0.98 reported in the main text is calculated for $\omega_0 = 3\ \Omega$, $\alpha = 11.1$, and $V = 0.5\ \Omega$ in the absence of decoherence and SPAM errors. For a static drive, the atoms would need an interaction strength of at least $V = 4.9\ \Omega$ to achieve the same fidelity.

Assuming a finite effective coherence time of 74 μs, the $|W\rangle$ fidelity under FFM reduces to 0.97 for $V = 0.5\ \Omega$. In this case, the corresponding interaction strength would have to be $V = 4.3\ \Omega$ for atoms under the static Rydberg excitation scheme to experience the same $|W\rangle$ fidelity (Supplementary Fig. 5). Nevertheless, since $(4.3/0.5)^{1/6} > \sqrt{2}$, it remains that for a square array, where nearest neighbors can be pairwise entangled using the static scheme, an atom can be pairwise entangled with its next-nearest neighbor with the same fidelity when driven by FFM.

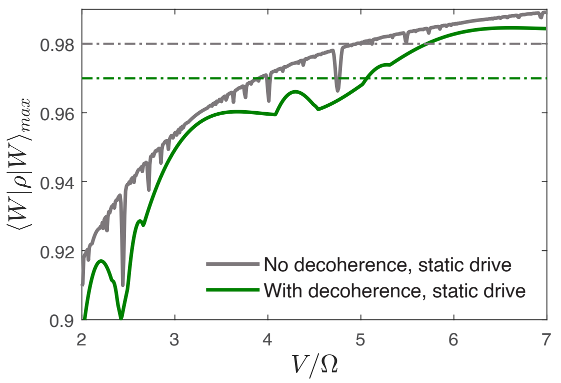

Supplementary Figure 5. **Comparison of maximum $|W\rangle$ fidelities achieved in a static Rydberg excitation against that from FFM.** Assuming no decoherence effects, FFM (ω_0 = 3 Ω, α = 11.1, V = 0.5 Ω) can yield (gray dot-dashed line) a fidelity of $\langle W|\rho|W\rangle_{\max} = 0.98$. To obtain the same fidelity in a static drive, atoms have to spaced more closely such that they experience (gray curve) V = 4.9 Ω. With a finite effective coherence time of 74 μs, the FFM fidelity decreases to (green dot-dashed line) 0.97. However, the atoms still have to experience an interaction strength of at least (green curve) V = 4.3 Ω for a static drive to achieve the same fidelity.

We note that the same effective extension of the Rydberg blockade range can be achieved in the static scheme with a lower Rabi frequency Ω_s, where $\Omega_s = \Omega J_0(\alpha)$. However, dynamic control of the Rydberg blockade range through FFM can potentially be simpler than that in the static scheme, especially where the atoms require two-color Rydberg excitation. In the latter case, accurately controlling a range of Rabi frequencies would require calibrating out the change in differential light shift that accompanies the nominal change in Rydberg laser intensities. Achieving the desired Rydberg laser intensities in turn requires a careful characterization of the AOM diffraction efficiency, which depends on both its RF drive frequency and amplitude. In contrast, FFM only requires the latter characterization of the AOM to realize a drive with minimal residual amplitude modulation. Further, the control of Rabi frequencies over a large range (0 to Ω) can already be achieved with a fairly small range of modulation indices (*e.g.* α = 2.4 to 0).

While this work focuses on using the ground state $|g\rangle$ and Rydberg state $|r\rangle$ as the two qubit states, one can apply the FFM scheme to entangle atoms that encode their qubit states in two (*e.g.* hyperfine) ground states, denoted here as $|0\rangle$ and $|1\rangle$. For instance, FFM is readily compatible with the Levine-Pichler protocol[8] that has been used to implement a two-qubit controlled-phase gate in the Rydberg blockade regime[9]. The conventional Levine-Pichler protocol uses two global Rydberg pulses of duration τ, sandwiched by a phase jump, to drive partial or full Rabi oscillations on two-qubit states with at least one qubit in $|1\rangle$. Where an extended Rydberg blockade range is desired, the global Rydberg pulses can be applied with a frequency-modulated detuning and with a user-defined modulation index α (Supplementary Fig. 6). We note, however, that as long as the interaction strength V is decreased, the two-qubit gate fidelity will be compromised, as shown by the following expression for the intrinsic two-qubit gate error[10] E_{min}:

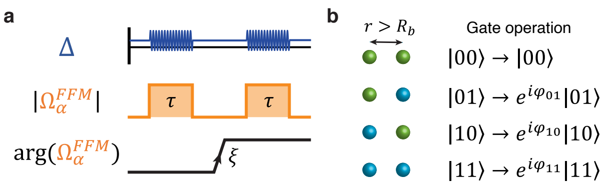

Supplementary Figure 6. **FFM-modified Levine-Pichler protocol[8] for implementing a controlled-phase gate. a** Where an extended Rydberg blockade range is desired, the two global Rydberg pulses of duration τ are applied with a frequency-modulated detuning given by $\Delta(t) = \Delta_0 + \delta\sin(\omega_0 t)$. **b** Even when two atoms are spaced farther than the blockade radius, the modified protocol can enable the two-qubit state to pick up a phase unless both qubits are in state $|0\rangle$.

$$E_{min} = \frac{3(7\pi)^{2/3}}{8}\frac{1}{(V\tau_R)^{2/3}} \quad (14)$$

where τ_R is the Rydberg state lifetime.

SUPPLEMENTARY NOTE 7: ENTANGLEMENT OF MULTIPLE ATOMS

For weak interactions $V < \Omega$, FFM can increase the effective Rydberg blockade radius relative to the static value, i.e. $R_b^{\rm FFM} > R_b$. Therefore, multiple atoms that reside within the increased blockade radius $R_b^{\rm FFM}$ can be entangled by driving them to the symmetric $|W\rangle$ state:

$$|W\rangle = \frac{1}{\sqrt{N}}\left(|egg\ldots g\rangle + |geg\ldots g\rangle + |gge\ldots g\rangle + \ldots\right) \quad (15)$$

As an example, we consider three atoms equally spaced on a line, where one of the atoms resides outside the static blockade radius of the first atom (Supplementary Fig. 7). Without FFM, the fidelity of the $|W\rangle$ state cannot exceed 0.80 for three atoms. However, with FFM, the fidelity is boosted to 0.99.

SUPPLEMENTARY NOTE 8: ANTIBLOCKADE THROUGH STIRAP

The steady-state population $|ee\rangle$ as shown in Fig. 4d of the main text can be achieved through the following STIRAP $\tanh$ profile:

$$\frac{\alpha(t)}{\alpha_0} = 1.2\cdot\tanh\left[-\frac{3.5}{T}\left(t-\frac{T}{2}\right)+0.23\right]+1\,, \quad (16)$$

where $\alpha_0 = 2.4$, such that $J_0(\alpha_0) = 0$, and T is the duration of the STIRAP sequence. The initial condition allows us to minimize the $|gg\rangle \leftrightarrow |W\rangle$ Rabi frequency while yielding a

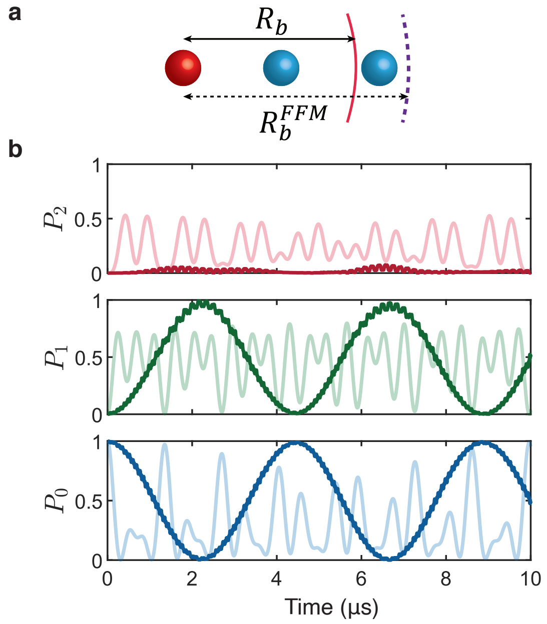

Supplementary Figure 7. **Increased entanglement range for multiple atoms.** **a** Three atoms are equally spaced in a chain. The interaction strength between next-nearest neighbors is $V = 0.5\,\Omega$, as indicated by the static blockade radius R_b (red curve). FFM is applied to extend the entanglement range to encompass next-nearest neighbors, as indicated by the extended blockade radius R_b^{FFM} (purple dashed curve). **b** Dynamics of three interacting atoms. P_n indicates the probability of exciting n atoms. For instance, P_1 maps onto the fidelity of the $|W\rangle$ state. (Solid curves) Under FFM ($\omega_0 = 6\,\Omega$, $\alpha = 11.2$), the three atoms can be driven to the entangled $|W\rangle$ state with high fidelity, where $P_1 = 0.99$. Such a high fidelity cannot be otherwise achieved with (light curves) a static drive.

reasonable $|W\rangle \leftrightarrow |ee\rangle$ coupling strength, whereas the final choice of α minimizes the $|W\rangle \leftrightarrow |ee\rangle$ Rabi frequency while giving the maximum $|gg\rangle \leftrightarrow |W\rangle$ coupling strength. The constants that appear in Supplementary Eq. (16) are chosen such that $J_0(\alpha(t=0)) = 0$, $J_1(\alpha(t=T)) = 0$ and $J_0(\alpha(t=T/2)) = J_1(\alpha(t=T/2))$.

We note that this STIRAP approach is generally robust against timing imperfections. Nevertheless, since the time required to do the STIRAP transfer is 4 μs and is comparable to our present coherence times, we did not attempt a demonstration of our proposed STIRAP scheme here.

We have pointed out in the main text that it is not possible to populate the $|ee\rangle$ state for two closely spaced atoms via STIRAP with the conventional monochromatic static excitation scheme. That said, one can adopt a version of STIRAP with two static frequency components. In this case, where each single-atom Rydberg excitation requires two colors, the bichromatic STIRAP scheme would require at least three lasers with individual control over each laser's detuning and intensity. In contrast, FFM is simpler to implement and does not require additional optomechanical components beyond what is needed for a monochromatic Rydberg drive.

SUPPLEMENTARY REFERENCES

[1] Aliyu, M. M., Zhao, L., Quek, X. Q., Yellapragada, K. C. & Loh, H. D 1 magic wavelength tweezers for scaling atom arrays. *Physical Review Research* **3**, 043059 (2021).

[2] Beterov, I. I., Ryabtsev, I. I., Tretyakov, D. B. & Entin, V. M. Quasiclassical calculations of blackbody-radiation-induced depopulation rates and effective lifetimes of rydberg ns, np, and nd alkali-metal atoms with $n \leq 80$. *Physical Review A* **79**, 052504 (2009).

[3] de Léséleuc, S., Barredo, D., Lienhard, V., Browaeys, A. & Lahaye, T. Analysis of imperfections in the coherent optical excitation of single atoms to rydberg states. *Physical Review A* **97**, 053803 (2018).

[4] Šibalić, N., Pritchard, J. D., Adams, C. S. & Weatherill, K. J. ARC: An open-source library for calculating properties of alkali rydberg atoms. *Computer Physics Communications* **220**, 319 (2017).

[5] Johansson, J. R., Nation, P. D., Nori, F. Qutip 2: A python framework for the dynamics of open quantum systems. *Computer Physics Communications* **184**, 1234 (2013).

[6] Levine, H. *et al.* High-fidelity control and entanglement of rydberg-atom qubits. *Physical Review Letters* **121**, 123603 (2018).

[7] Tamura, H., Yamakoshi, T. & Nakagawa, K. Analysis of coherent dynamics of a rydberg-atom quantum simulator. *Physical Review A* **101**, 043421 (2020).

[8] Levine, H. *et al.* Parallel implementation of high-fidelity multiqubit gates with neutral atoms. *Physical Review Letters* **123**, 170503 (2019).

[9] Jaksch, D. *et al.* Fast quantum gates for neutral atoms. *Physical Review Letters* **85**, 2208 (2000).

[10] Saffman, M. Quantum computing with atomic qubits and rydberg interactions: progress and challenges. *Journal of Physics B: Atomic, Molecular and Optical Physics* **49**, 202001 (2016).
